# Chloroplast genome data of five *Amygdalus* species: Clarifying genome structure and phylogenetic relationships

**DOI:** 10.1016/j.dib.2024.110077

**Published:** 2024-01-24

**Authors:** Yixiao Chen, Wenquan Bao, Dun Ao, Yue Bai, Haiguang Huang, Rong Yang, Lin Wang, Ta-na Wuyun

**Affiliations:** a Inner Mongolia Agricultural University, 010000, Hohhot, China; b State Key Laboratory of Tree Genetics and Breeding, Research Institute of Non-Timber Forestry, Chinese Academy of Forestry, 450003, Zhengzhou, China; c Inner Mongolia Academy of Forestry Science, 010000, Hohhot, China

**Keywords:** *Amygdalus*, Comparative genomics, Sequence divergence, Phylogenetic relationships

## Abstract

*Amygdalus* species have considerable ecological and economic value, however, the phylogenetic relationships among *Amygdalus* remain controversy. In this study, we sequenced and assembled the chloroplast (cp) genomes of five *Amygdalus* species: *Prunus communis, P. mongolica, P. pedunculata, P. triloba*, and *P. mira*. We then conducted comparative genomic analyses and constructed their phylogenetic relationships. The genome length ranged from 157,870 to 158,451 bp, and 131 genes were annotated (86 protein-coding genes, 37 tRNAs, and 8 rRNAs). Additionally, 49–57 simple sequence repeats were detected, with most in the large single-copy region and with AT base preferences. Comparative genomic analyses revealed high similarities in structure, order, and gene content. However, we identified four highly divergent sequences: *trnR-UCU*-*atpA, nbdhC*-*trnV-UAC, ycf4*-*cemA*, and *rpl32*-*trnL-UAG*. The phylogenomic relationship analysis suggested that the *Amygdalus* species were grouped together, in which *P. pedunculata, P. triloba*, and *Prunus tangutica* were categorized into a branch, *P. mongolica* and *Prunus davidiana* were clustered a branch. This study provides an improved understanding of the genetic relationships among the *Amygdalus* and provides a basis for the development and utilization of *Amygdalus* resources.

Specifications Table

**Table:** 

Subject	Biological sciences
Specific subject area	Omics: Genomics
Data format	Raw Analyzed
Type of data	Table Figure
Data collection	An Illumina Hiseq X high-throughput platform (Illumina, San Diego, CA, USA) was used for DNA sequencing. Chloroplast genome fragments were assembled using SOAPdenovo (http://soap.genomics.org.cn/soapdenovo.html) to obtain contigs and optimized assembly results according to the reference cp genome of *Prunus persica* (GenBank accession number NC_014697.1). Genomes were annotated using DOGMA (http://dogma.ccbb.utexas.edu/). An annotated cp genome map was constructed with Organellar Genome Draw (https://chlorobox.mpimp-golm.mpg.de/OGDraw.htmL).
Data source location	• Institution: Non-timber Forest Research and Development Center, Chinese Academy of Forestry • City: Zheng zhou • Country: China
Data accessibility	Repository name: Mendeley Data Data identification number: DOI:10.17632/kh83zb2f9p.1 Direct URL to data: https://data.mendeley.com/datasets/kh83zb2f9p/1

## 1 Value of the Data

• Chloroplast genome sequences of five Amygdalus species provides an improved understanding of the genetic relationships among the Amygdalus.
• Identification of these SSR loci and variations provides candidate molecular markers for research on population diversity and evolutionary research.
• The chloroplast genome data provides a basis for the development and utilization of Amygdalus resources.

## 2 Data Description

The genus *Amygdalus* was classified into subgenus *Amygdalus* and subgenus *Persica* [1]. The subgenus *Amygdalus* is mainly distributed in the Mediterranean region and central-eastern Asia, with the exception of *P. triloba*, which widely distribution in northwest China [2]. The *P. triloba, P. pedunculata, P. mongolica*, and *P. communis* belonged to subgenus *Amygdalus,* and the kernels were rich in oil and protein, which can be use as high-quality oil and protein resource [3,4]. The shell can also be used as fuel and adsorbent for heavy metals and pigments [5]. *P. mira*, belonging to subgenus *Persica*, is mainly distributed in the Yarlung Zangbo Grand Canyon and the tributary basins of Tibetan Plateau [6]. The kernels of *P. mira* are rich in oleic acids, linoleic acids, and fat-soluble components, which was used for Chinese traditional medicine to treatment and improvement of diseases [7].

The classification of genus *Amygdalus* has always been controversial [8]. Base on the morphological classification, the *P. communis, P. mongolica, P. tangutica, P. triloba*, and *P. pedunculata* were classified into subgenus *Amygdalus*, whereas, the *P. mira, P. davidiana, P. ferganensis, P. kansuensis*, and *P. persica* were classified into subgenus *Persica* [1]. However, Wang et al. [9] revealed that *Prunus tenella, P. pedunculata*, and *P. triloba* should be classified into the genus *Prunus L*., while, *P. communis, P. mongolica*, and *P. tangutica* be divided into the subgenus *Amygdalus L*. by using cp genomes, and Yazbek et al. [10] investigated the phylogeny of *Prunus subg. Amygdalus* by plastid and nuclear genes found that the *P. triloba*, and *P. pedunculata* should be excluded from subgenus *Amygdalus*.

Chloroplasts (cp) are semi-autonomous organelles with a conserved, maternally inherited genome separate from the rest of the plant. The genome size of terrestrial plants cp varies from 100 to 200 kb and generally contains 110–130 genes, which are mainly composed of genes involved in photosynthesis, transcription, and translation [11]. Most higher-plant cp genomes form a quadripartite structure, containing one pair of inverted repeats (IR), a small single-copy (SSC) region, and a large single-copy (LSC) region separated by IRs [12]. Therefore, the study of the cp genome plays an important role in species identification, phylogenetic analysis, and molecular marker development [13,14]. The rapid development of next-generation sequencing technologies and phylogenetic genomics has led to the sequencing of cp genomes from multiple *Prunoideae* species, which are widely used in molecular evolution and phylogenetic research [15], [16], [17]. However, the cp genomes of *Amygdalus* species were remain insufficient. Here, we sequenced, assembled, and performed phylogenetic analysis on the complete cp genomes of five *Amygdalus* species (*P. communis, P. mongolica, P. pedunculata, P. triloba*, and *P. mira*) aimed to provide an improved understanding of the genetic relationships among the Amygdalus and provide a basis for the development and utilization of *Amygdalus* resources.

**Fig. 1:**
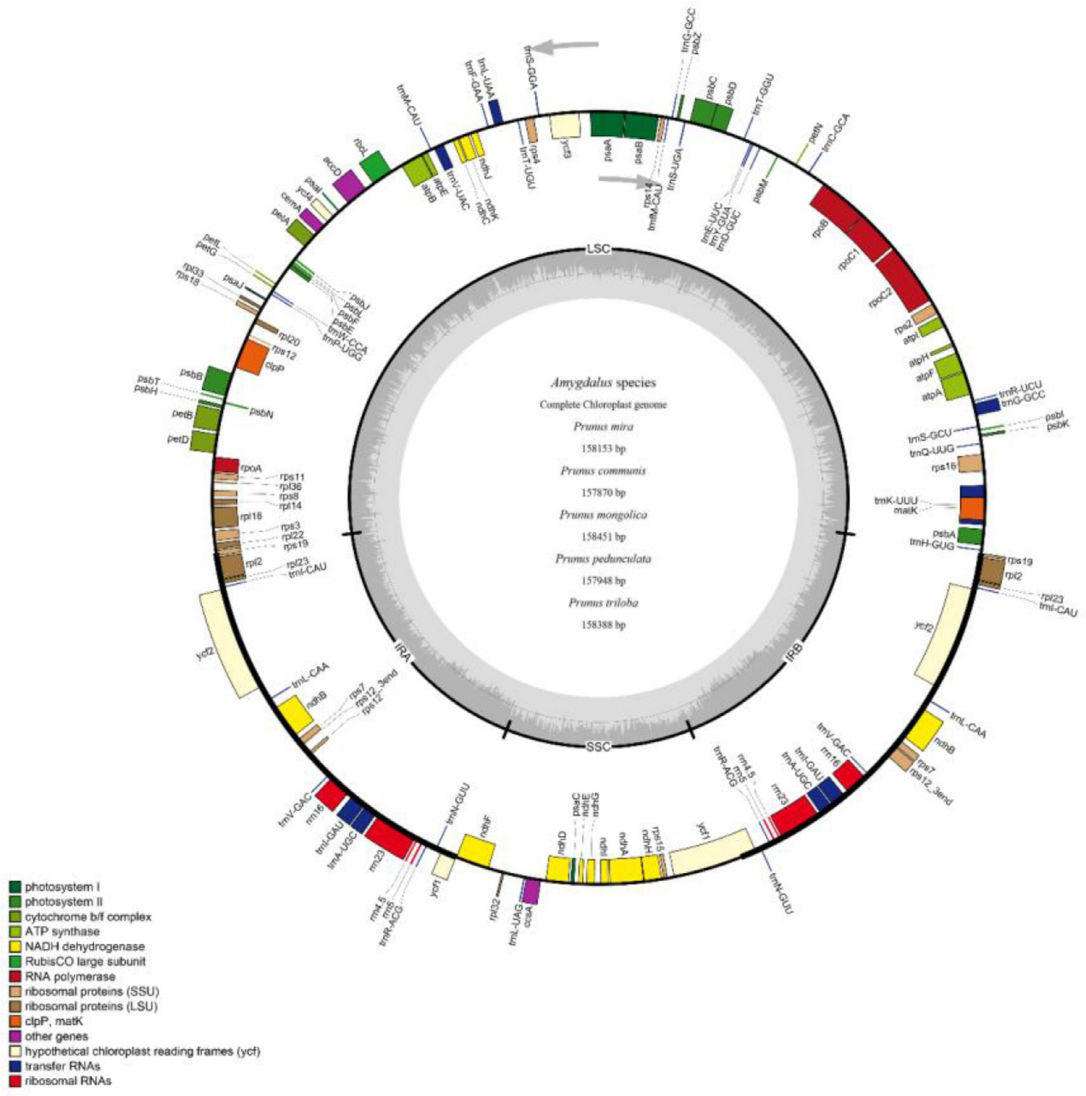
Assembly, size, and features of the chloroplast genomes from five *Amygdalus* species. The dark gray area in the inner circle represents genomic GC content, whereas the light gray area indicates AT content.

Total cp genome lengths for *P. mira, P. communis, P. mongolica, P. pedunculata*, and *P. triloba* were 158,153, 157,870, 158,451, 157,948, and 158,388 bp, respectively (Fig. 1). Each cp genome exhibited a typical quadripartite structure, including two IRs (26,373–26,931 bp), one LSC (86,144–86,525 bp), and one SSC (18,966–19,211 bp). The GC content in the cp genome ranged from 36.72 % in *P. mongolica* to 36.78 % in *P. pedunculata* and was higher in the IR regions (ranging from 42.55 % in *P. mira* to 42.60 % in *P. communis*) than that in the LSC regions (ranging from 34.57 % in *P. communis* to 34.62 % in *P. pedunculata*) and the SSC regions(ranging from 30.27 % in *P. mongolica* to 30.46 % in *P. communis*), suggesting that the two IR regions were relatively stable.

We annotated 131 genes in each of the five *Amygdalus* cp genomes, including 86 protein-coding genes, 37 transfer RNAs (tRNA), and eight (rRNAs) (Table 1). These genes were in the same order across all five cp genomes.

**Table 1:** Chloroplast genomes of the five *Amygdalus* species.

Species	Genome	G-C Content(%)				Genes	CDS	tRNA	rRNA
		Genome	LSC	SSC	IR				
*Prunus mira*	158,153	36.74	34.6	30.35	42.55	131	86	37	8
*Prunus communis*	157,870	36.76	34.57	30.46	42.6	131	86	37	8
*Prunus mongolica*	158,451	36.72	34.58	30.27	42.58	131	86	37	8
*Prunus pedunculata*	157,948	36.78	34.62	30.45	42.59	131	86	37	8
*Prunus triloba*	158,388	36.77	34.62	30.44	42.59	131	86	37	8

Functional analysis classified 131 genes into four categories, including photosynthesis-related, self-replication-related, other, and unknown function(Table 2). A total of 18 genes were duplicated in the IR region. Furthermore, 18 contained one intron each, while two genes (*clpP* and *ycf3*) contained two introns (Table S1). In addition, the GC content of rRNA (55.5 %) and tRNA (ranging from 53.3 % in *P. communis* to 53.41 % in *P. mira*) was higher than that in protein-coding genes (ranging from 37.61 % in *P. triloba* to 37.65 % in *P. communis*) (Table S2).

**Table 2:** Genes identified in the chloroplast genomes of the five *Amygdalus* species.

Gene categories	Gene groups	Gene names
Self-replication	Ribosomal RNAs	*rrn16*^b^, *rrn23*^b^, *rrn4.5*^b^, *rrn5*^b^
	Transfer RNAs	*trnA-UGC^a,b^, trnC-GCA, trnD-GUC, trnE-UUC, trnF-GAA, trnfM-CAU, trnG-GCC^a^, trnH-GUG, trnI-CAU^b^, trnI-GAU^a,b^, trnK-UUU^a^, trnL-CAA^b^, trnL-UAA^a^, trnL-UAG, trnM-CAU, trnN-GUU^b^, trnP-UGG, trnQ-UUG, trnR-ACG^b^, trnR-UCU, trnS-GCU, trnS-GGA, trnS-UGA, trnT-GGU, trnT-UGU, trnV-GAC^b^, trnV-UAC^a^, trnW-CCA, trnY-GUA*
	Proteins of small ribosomal subunit	*rps2, rps3, rps4, rps7*^b^, *rps8, rps11, rps12^a,^*^b^, *rps14, rps15, rps16*^a^, *rps18, rps19^b^*
	Proteins of large ribosomal subunit	*rpl2*^a, b^, *rpl14, rpl16*^a^, *rpl20, rpl22, rpl23*^b^, *rpl32, rpl33, rpl36*
	Subunits of RNA polymerase	*rpoA, rpoB, rpoC1*^a^, *rpoC2*
Genes for photosynthesis	Subunits of NADH-dehydrogenase	*ndhA*^a^, *ndhB*^a b^, *ndhC, ndhD, ndhE, ndhF, ndhG, ndhH, ndh*I, *ndhJ, ndhK*
	Subunits of Photosystem Ⅰ	*psaA, psaB, psaC, psaI, psaJ*
	Subunits of Photosystem Ⅱ	*psbA, psbB, psbC, psbD, psbE, psbF, psbH, psbI, psbJ, psbK, psbL, psbM, psbN, psbT, psbZ*
	Large subunit of RuBisCO	*rbcL*
	Subunits of cytochrome b/f complex	*petA, petB^a^, petD^a^, petG, petL, petN*
	Subunits of ATP synthase	*atpA, atpB, atpE, atpF*^a^, *atpH, atpI*
Other genes	Acetyl-CoA carboxylase	*accD*
	Cytochrome c biogenesis	*ccsA*
	Maturase	*matK*
	Envelope membrane protein	*cemA*
Unknown	Conserved hypothetical chloroplast reading frames	*ycf1*^b^, *ycf2*^b^, *ycf3*^a^, *ycf4*

**Fig. 2:**
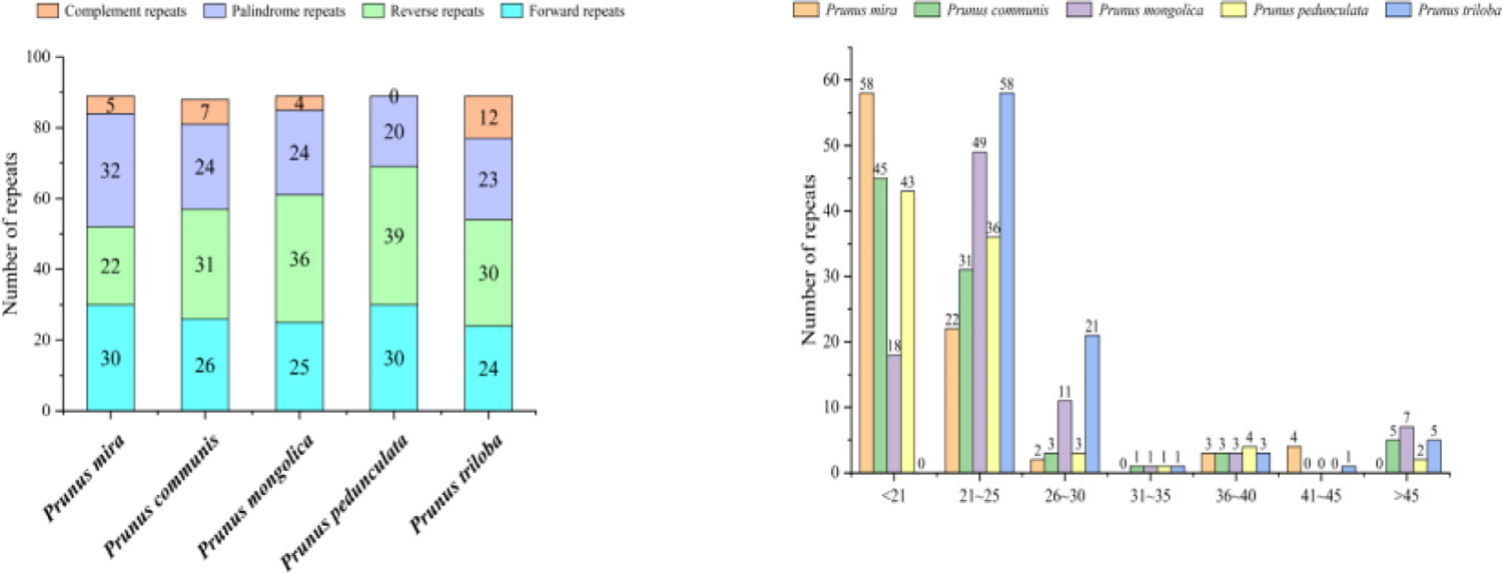
(A.left) Number of dispersed repeats in the five *Amygdalus* species. (B.right) Number of long repeat sequences, clustered by length, in the five *Amygdalus* species.

We identified 89 repeat sequences, including forward, reverse, palindromic, and complement repeats in *P. mira, P. mongolica, P. triloba*, and *P. pedunculata*, while *P. communis* had 88 repeat sequences (Fig. 2). The lengths of the repeat sequence in *P. mira, P. mongolica, P. communis*, and *P. pedunculata* was 18–25 bp, while that in *P. triloba* was 21–30 bp. Five, seven, four, and twelve complement repeats were found in *P. mira, P. communis, Prunus mongolica*, and *P. triloba*, respectively. Reverse repeats (31, 36, 39, 30) were more abundant than palindromic repeats (24, 24, 20, 23) in *P. communis, P. mongolica, P. pedunculata*, and *P. triloba*, respectively. In contrast, *P. mira* had fewer reverse repeats (22) than palindromic repeats (32).

**Fig. 3:**
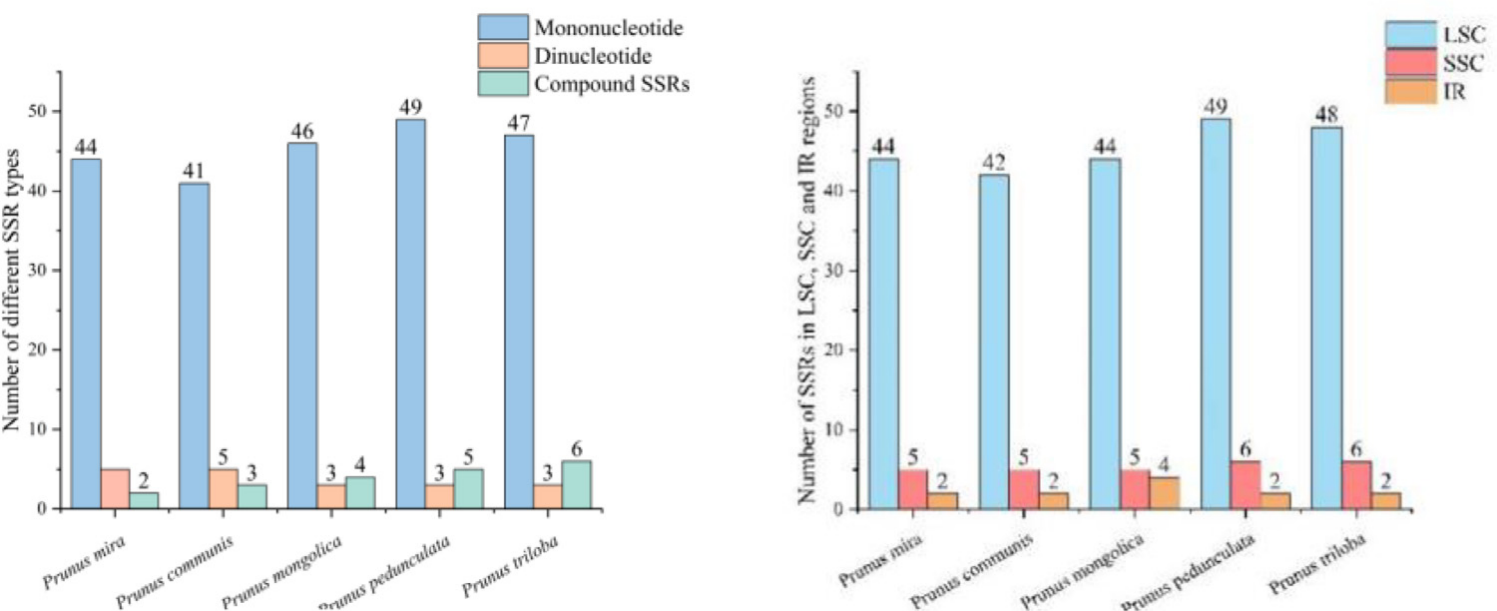
(A.left) Number and type of short sequence repeats (SSRs). B. right) Number of SSRs in the large single-copy (LSC), small single-copy (SSC), and inverted-repeat (IR) regions.

Three types of SSRs (mononucleotide, dinucleotide, and compound) were present in the five *Amygdalus* cp genomes (Fig. 3a), in counts ranging from 49 (*P. communis*) to 57 (*P. pedunculata*). Mononucleotide repeats (ranging from 83.67 % in *P. communis* to 86.79 % in *P. mongolica*) were the most frequently occurring, followed by dinucleotide (ranging from 5.26 % in *P. pedunculata* to 10.20 % in *P. communis*) and compound SSRs (ranging from 3.92 % in *P. mira* to 10.71 % in *P. triloba*). The number of A (ranging from 29.27 % in *P. communis* to 40.91 % in *P. mira*) and T (ranging from 50.00 % in *P. mira* to 60.98 % in *P. communis*) mononucleotide repeats was higher than that of C (4.35 % in *P. mongolica* to 8.51 % in *P. triloba*) or G repeats (4.08 % in *P. pedunculata* to 4.88 % in *P. communis*) (Table S3). and two dinucleotide repeat motifs were obtained: AT (ranging from 3.51 % in *P. pedunculata* to 5.88 % in *P. mira*) and TA (ranging from 1.75 % in *P. pedunculata* to 6.12 % in *P. communis*).

We further analyzed the distribution of SSRs (Fig. 3b) and found that most SSRs were distributed in the LSC regions (83.02–86.27 %); fewer were in the SSC regions (9.43–10.71 %) and IR regions (3.51–7.55 %) regions. Additionally, 79.59–84.21 % SSRs were located in intergenic spacer (IGS) regions, while 15.79–20.41 % SSRs were in coding sequences (CDS).

**Fig. 4:**
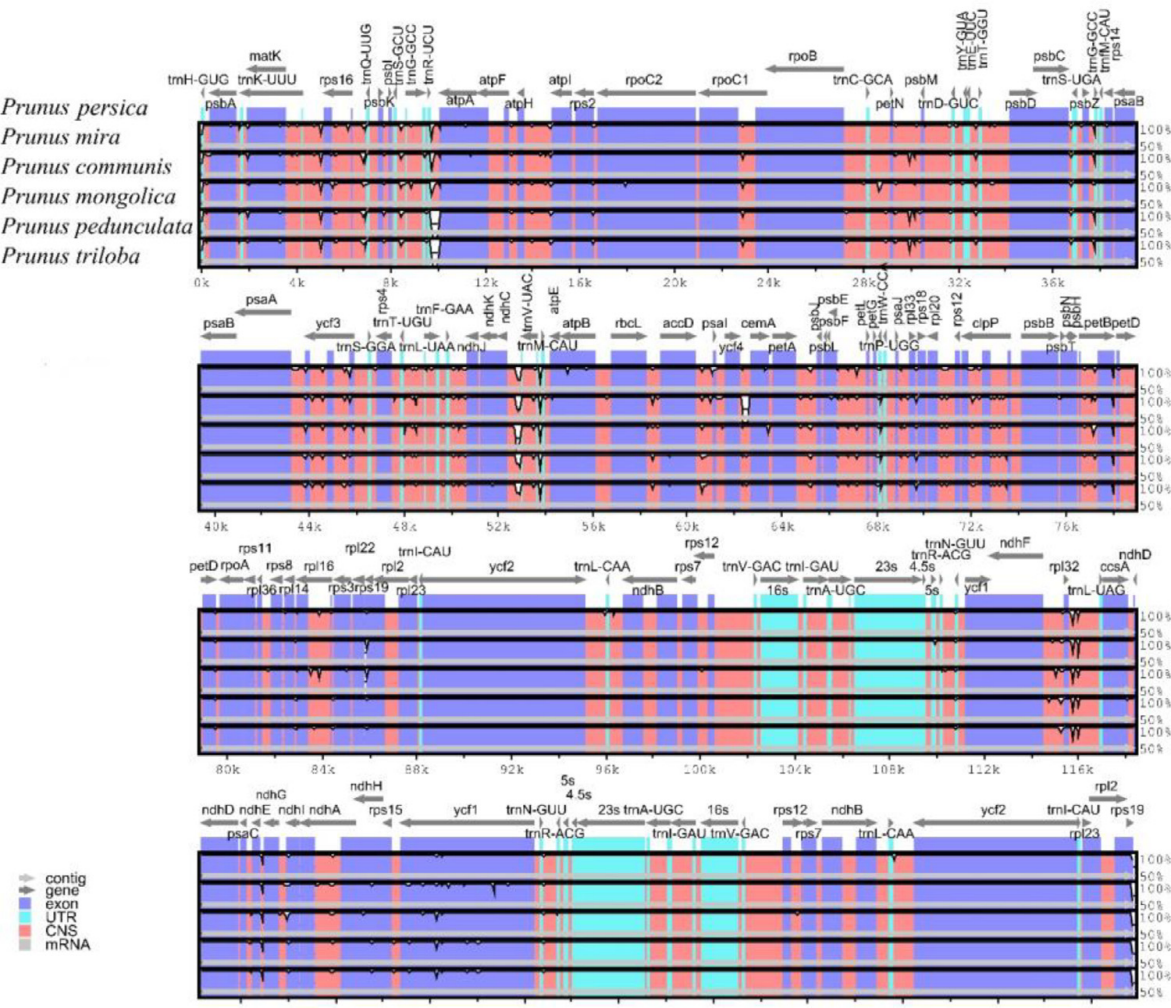
Sequence alignment of chloroplast genomes from the five *Amygdalus* species. The y-axis represents percent identity from 50 to 100 %.

The analysis of cp genome sequence alignment of five *Amygdalus* species were carried out by using *P. persica* as a reference, the result showed that the variations in IGS were higher than that in CDS (Fig. 4). We identified four highly divergent sequences in the IGS: *trnR-UCU*-*atpA, nbdhC*-*trnV-UAC, ycf4*-*cemA*, and *rpl32*-*trnL-UAG*. These sequences have potential candidate molecular markers for *Amygdalus* species.

**Fig. 5:**
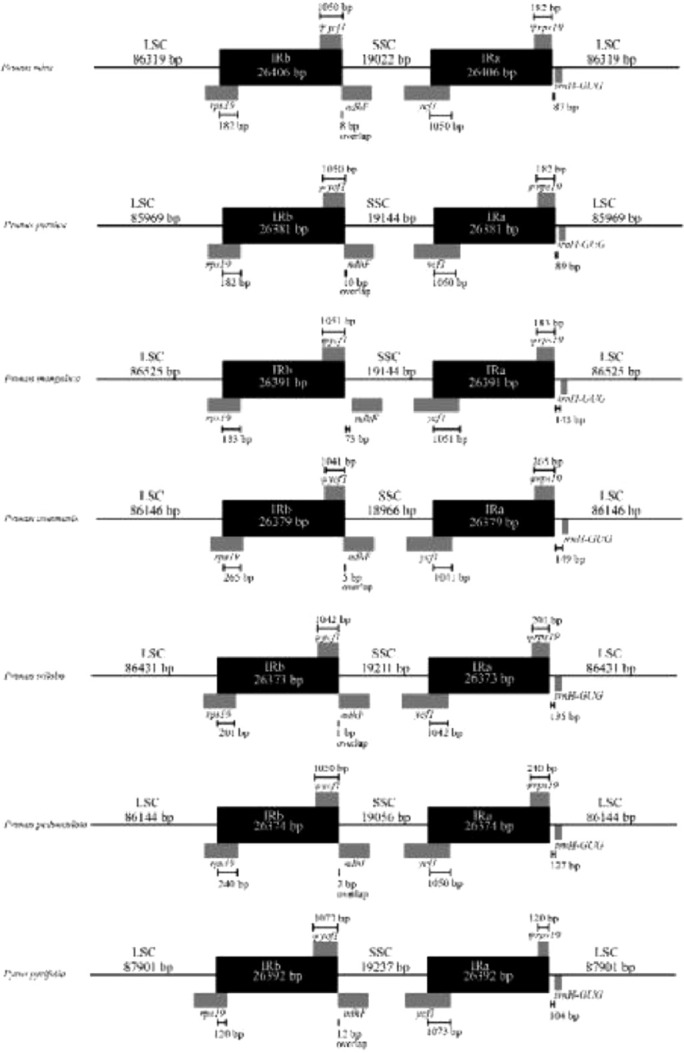
Comparison of LSC, SSC, and IR border regions across the five *Amygdalus* species.

We compared IR regions among the cp genomes of the five *Amygdalus* species and two related species (*P. persica* and *P. pyrifolia*) (Fig. 5). Although the cp genome structure and gene organization were highly conserved, IR expansions and contractions resulted in slight variations in the LSC/IRb and SSC/IRa borders. The genes of *rps19, ycf1*, and *ndhF* were distributed near the boundaries of IR/LSC and IR/SSC. Among them, the *ycf1* was detected at the LSC/IRa boundary and the size of *ycf1* in the IRa region was 1041–1051 bp, the *rps19* was located at the LSC/IRb boundary, with a fragment size of 182–265 bp in the IRb region. The *ndhF* was located at the IRb/SSC border with a fragment size of 1, 2, 5, and 8 bp in *P. triloba, P. pedunculata, P. communis*, and *Prunus Mira*, respectively, however, the *ndhF* in *P. mongolica* did not overlap at IRb/SSC boundary.

**Fig. 6:**
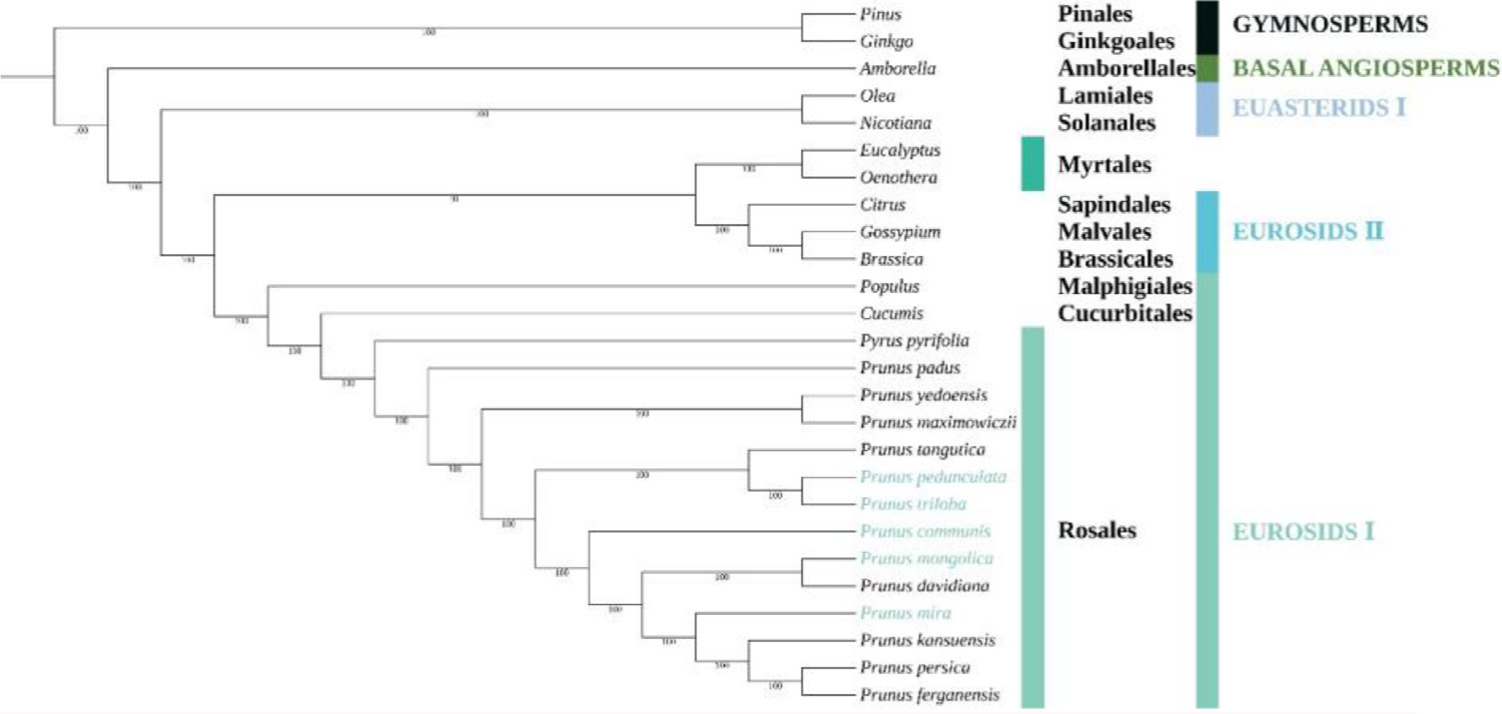
Maximum-likelihood (ML) phylogenetic tree of 26 species based on complete chloroplast sequences. The bootstrap values are marked at the tree node.

Our phylogenetic trees based on complete cp genome data had a higher bootstrap value, and 23 out of 24 nodes had 100 % bootstrap values (Fig. 6).

To further ascertain the phylogenetic position of *Amygdalus*, a phylogenetic analysis was carried out among 26 species. The result showed that five groups were divided, including eurosids I, eurosids II, gymnosperms, basal angiosperms, and euasterids I (Fig. 6, Table S4). The phylogenomic relationship analysis suggested the *Amygdalus* species were grouped together. Furthermore, four *Persica* species*,* including *P. mira, Prunus kansuensis, Prunus ferganensis*, and *P. persica* were clustered into a separate branch, and *P. pedunculata, P. triloba*, and *Prunus tangutica* were categorized into a branch, while *P. mongolica* and *Prunus davidiana* were clustered a branch, these results are consistent with previous reports [2,17].

## 3 Experimental Design, Materials and Methods

Young and fresh leaves were obtained from all five species. *P. mira* samples were collected from Bengga, Tibet; *P. pedunculata* and *P. triloba* were collected from Inner Mongolia; *P. mongolica* and *P. communis* were collected from the Gansu and Henan Provinces, respectively. Samples were stored at −80 °C until analysis.

Total genomic DNA was extracted using a Plant Genomic DNA Kit (Tiangen, Beijing, China). An Illumina Hiseq X high-throughput platform (Illumina, San Diego, CA, USA) was used for DNA sequencing. Library preparation and sequencing were completed by BGI Genomics (Shenzhen, China).

Chloroplast genome fragments were assembled using SOAPdenovo (http://soap.genomics.org.cn/soapdenovo.html) to obtain contigs and optimized assembly results according to the reference cp genome of *Prunus persica* (GenBank accession ID NC_014697.1). Spaces between long contigs were modified using Gapcloser (https://sourceforge.net/projects/soapdenovo2/files/GapCloser/) to obtain a complete cp genome.

Genomes were annotated using DOGMA (http://dogma.ccbb.utexas.edu/), and the annotation results were manually modified using Geneious (https://www.geneious.com/). An annotated cp genome map was constructed with Organellar Genome Draw (https://chlorobox.mpimp-golm.mpg.de/OGDraw.htmL).

Genomic GC/AT content, as well as lengths of LSC, SSC, IRa, IRb, intergenic regions, exons, introns, tRNA, and rRNA, were counted in DNASTAR (https://www.dnastar.com/).

CpGAVAS (http://124.17.107.12/0506/cpgavas/analyzer/home) was used to visualize the location and length of IR boundaries and junctions.

Long repeat sequences, including forward, palindromic, reverse, and complement repeats, were identified using REPuter (http://bibiserv.techfak.uni-biele.org.de/reputer/); the length and similarity of these sequences were more than 15 bp and 90 %, respectively. The distribution of simple sequence repeats (SSRs) (mono-, di-, tri-, tetra-, penta-, and hexanucleotide repeats) was predicted using MISA (https://webblast.ipk-gatersleben.de/misa/index.php), with a minimum number of six, four, four, three, three and three, respectively. Compound SSRs were those interrupted by a nonrepetitive nucleotide sequence.

Sequences were aligned using mVISTA (http://genome.lbl.gov/vista/index.shtml) to analyze homology and similarity.

The LSC, SSC, and IR border sequences in *Amygdalus* cp genomes were compared against those in the cp genomes of *P. persica* (NC_014697) and *Pyrus pyrifolia* (NC_015996). The IR-SC boundaries of the cp genomes were visualized in IRscope.

The cp genome sequences of 24 angiosperms and 2 gymnosperms were selected for phylogenetic analysis. Multiple genome sequences were aligned in MAFFT version 7 (https://www.ebi.ac.uk/Tools/msa/mafft/). *Pinus thunbergia* (NC_001631) and *Ginkgo biloba* (NC_016986) were chosen as outgroups. The maximum likelihood (ML) phylogenetic tree was constructed based on the complete chloroplast genome sequences using the GTAGAMMA model with a bootstrap test of 1000 replicates in RAxML version 8.2.12 (CIPRES Science Gateway version 3.3, https://www.phylo.org/). Trees were visualized using iTOL (https://itol.embl.de/).

## Limitations

None.

## Ethics Statement

All authors have read and follow the ethical requirements for publication in Data in Brief and confirming that the current work does not involve human subjects, animal experiments, or any data collected from social media platforms.

## CRediT authorship contribution statement

**Yixiao Chen:** Methodology, Investigation, Writing – original draft. **Wenquan Bao:** Writing – review & editing. **Dun Ao:** Formal analysis, Resources. **Yue Bai:** Formal analysis, Resources. **Haiguang Huang:** Formal analysis, Resources. **Rong Yang:** Formal analysis, Resources. **Lin Wang:** Writing – review & editing. **Ta-na Wuyun:** Conceptualization.

## Data Availability

Chloroplast Genome data of Five Amygdalus Species (Original data) (Mendeley Data)

## Appendix Supplementary materials

Image, application 1

Image, application 2

Image, application 3

Image, application 4
